# An interdisciplinary intervention for older Taiwanese patients after surgery for hip fracture improves health-related quality of life

**DOI:** 10.1186/1471-2474-11-225

**Published:** 2010-09-29

**Authors:** Yea-Ing L Shyu, Jersey Liang, Chi-Chuan Wu, Huey-Shinn Cheng, Min-Chi Chen

**Affiliations:** 1 School of Nursing, Chang Gung University, 259 Wen-Hwa 1st Road, Kwei-Shan, Taoyuan 333, Taiwan; 2 School of Public Health, University of Michigan, 1420 Washington Heights, M3234, SPH II, Ann Arbor, MI 48109-2029, USA; 3 Institute of Gerontology, University of Michigan, 300 North Ingalls, 9th Floor, Ann Arbor, MI 48109-2007, USA; 4 Traumatological Division, Department of Orthopedics, Chang Gung Memorial Hospital, 5 Fu-Hsin Street, Kwei-Shan, Taoyuan 333, Taiwan; 5 Department of Internal Medicine, Chang Gung Memorial Hospital, 5 Fu-Hsin Street, Kwei-Shan, Taoyuan 333, Taiwan; 6 Department of Public Health & Biostatistics Consulting Center, Chang Gung University, 259 Wen-Hwa 1st Road, Kwei-Shan, Taoyuan 333, Taiwan

## Abstract

**Background:**

The effects of intervention programs on health-related quality of life (HRQOL) of patients with hip fracture have not been well studied. We hypothesized that older patients with hip fracture who received our interdisciplinary intervention program would have better HRQOL than those who did not.

**Methods:**

A randomized experimental design was used. Older patients with hip fracture (N = 162), 60 to 98 years old, from a medical center in northern Taiwan were randomly assigned to an experimental (n = 80) or control (n = 82) group. HRQOL was measured by the SF-36 Taiwan version at 1, 3, 6, and 12 months after discharge.

**Results:**

The experimental group had significantly better overall outcomes in bodily pain (β = 9.38, p = 0.002), vitality (β = 9.40, p < 0.001), mental health (β = 8.16, p = 0.004), physical function (β = 16.01, p < 0.001), and role physical (β = 22.66, p < 0.001) than the control group at any time point during the first year after discharge. Physical-related health outcomes (physical functioning, role physical, and vitality) had larger treatment effects than emotional/mental- and social functioning-related health outcomes.

**Conclusions:**

This interdisciplinary intervention program may improve health outcomes of elders with hip fracture. Our results may provide a reference for health care providers in countries using similar programs with Chinese/Taiwanese immigrant populations.

**Trial registration:**

NCT01052636

## Background

Hip fracture is a serious health problem in the elderly because it leads to excess mortality of 5-20%, and morbidity that severely impedes health-related quality of life (HRQOL) for patients [1-4]. With an increasingly aging population [5], hip fracture represents a major and growing health care problem in Taiwan as in many other countries [6].

Elderly patients with hip fracture have been found to benefit from postoperative rehabilitation, rehabilitation on an ortho-geriatric unit, early discharge planning programs, transitional care programs, or extended outpatient rehabilitation [7-13]. Traditional indicators of disease and treatment outcomes such as mortality and objective clinical parameters have been supplemented by measures of self-rated health-related quality of life (HRQOL) [14,15], which has been suggested as a measure for patients with hip fracture [16]. However, interventions to improve the HRQOL of elders with hip fracture have had inconsistent results. For example, elders' HRQOL was reported to improve within 6 months of discharge after receiving discharge planning or a home-based intervention [13,17,18]. On the other hand, HRQOL was reported to improve little or not all in other interventional studies [12,19,20].

At the same time, the vast majority of interventional studies for hip-fractured elders were conducted in Western developed countries and few of them used data from more than two time points to examine the longitudinal effects of interventions up to 1 year after discharge. Little is known about the effects, specifically the long-term effects, of intervention programs on HRQOL for elderly patients with hip fracture in Chinese populations.

The short- and long-term effects of an interdisciplinary intervention program for elders with hip fracture in Taiwan were examined by our group in a previous randomized experimental study [21,22]. The interdisciplinary program consisted of geriatric consultation, continuous rehabilitation, and discharge planning. We found that the intervention program may benefit elders with hip fracture in Taiwan by improving their HRQOL within 3 months after discharge. The purpose of this paper is to report the long-term effects of the intervention program on HRQOL of hip-fractured elders within 1 year after discharge. We hypothesized that subjects who received the interdisciplinary intervention would have better HRQOL than controls throughout the first year after discharge.

## Methods

### Participants

Inclusion criteria for subjects were (1) ≥ 60 years, (2) admitted to hospital for an accidental single-side hip fracture, (3) receiving hip arthroplasty or internal fixation, (4) able to perform active movement against gravity and some resistance or full resistance, and with pre-fracture Chinese Barthel Index (CBI) score > 70, and (5) living in northern Taiwan. Patients were excluded if they were (1) severely cognitively impaired and completely unable to follow orders (determined by a score < 10 on the Chinese Mini-Mental State Examination [MMSE, 23]), or (2) terminally ill.

Muscle power of the unaffected limb at admission (post hip fracture) was assessed by a trained research nurse. The pre-fracture CBI score was obtained from participants or/and carers by the research nurse. Using the Barthel Index to retrospectively assess pre-fracture physical functioning has been suggested by the UK National Health Service [24]. Although outcomes of cognitively impaired elders have been improved by intensive rehabilitation programs [25-27], this study included only participants with mild and moderate cognitive impairment. This decision was based on our reasoning that those with severe cognitive impairment (MMSE score < 10) are disoriented to time, place, and persons, have lost their learning ability due to severe memory deficit, and have difficulty following directions [28], which might require different protocols for the rehabilitation intervention. To avoid complicating the study design, we included only participants with mild and moderate cognitive impairment.

**Figure 1:**
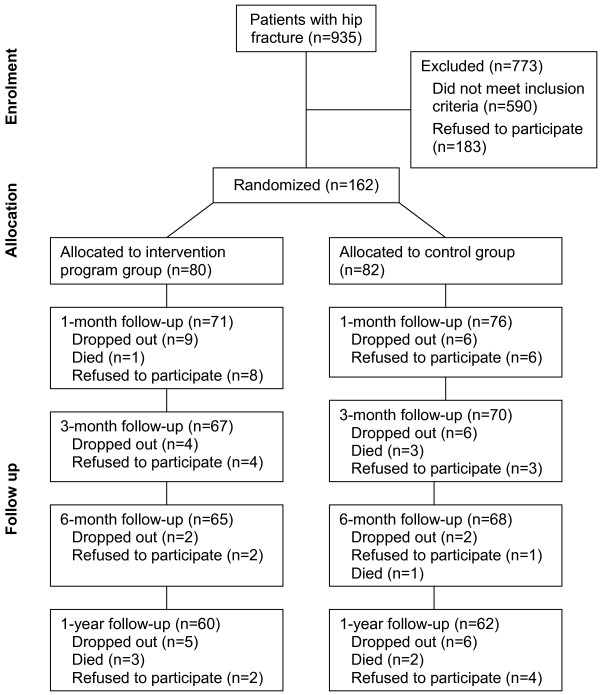
**Study flow diagram**. Sample recruitment process.

The sample was recruited and followed from September 2001 to November 2003 (Figure 1). Of those who met the criteria and agreed to participate (n = 162), 80 were randomly assigned to the experimental, and 82 to the control group. Patients who declined to participate (n = 134) and those who agreed (n = 164) did not differ significantly in age (p = 0.322) and gender (p = 0.517). Based on our pilot study data, a power of 0.80, and an alpha of 0.05, we estimated a sample size of 65 subjects in each group to obtain a median effect size of 0.50 [29] for improved performance of activities of daily living (ADLs) measured by the CBI (experimental vs control = 38.5 vs 31.5) and 61 subjects in each group for improved physical functioning measured by the physical function scale of the Taiwan version of the Medical Outcomes Study Short-Form 36 (SF-36) [30] from post-surgery to the third month after discharge (experimental vs control = 22.9 vs 11.1) [21,22]. To allow for 18% to 24% potential dropouts, we therefore aimed to recruit around 80 subjects in each group.

### Routine care (control group)

Current practice in caring for elders with hip fracture in Taiwan lacks well-organized interdisciplinary care protocols and continuity of care. After a fall incident, patients usually are sent directly to the hospital emergency room (ER) and are cared for by orthopedists. Elderly patients with a femoral neck fracture of subcapital and transcervical types are treated with hip hemiarthroplasty and the basal neck type is treated with closed reduction using dynamic hip screw fixation. Before surgery, referrals are occasionally made for internal medicine care. Routine examinations before surgery include EKG, blood chemistry tests and cell counts, and X-rays. In the first 2 to 3 days after surgery, patients receive antibiotics and pain medication, and are taught to exercise with caution while still in bed. Physical therapy usually starts on the second or third day after removal of the hemovac, which is used for postoperative wound drainage. The usual hospital stay following surgery is around 7 days and no in-home programs are provided. Adherence to clinical follow-ups at 1, 3, 6 and 12 months after surgery is poor. Patients are encouraged to ambulate with protected weight bearing for 3 months. Using a walker and touching the ground lightly are recommended.

### Intervention program (experimental group)

The intervention program developed for this study included three components: geriatric consultation service, rehabilitation program, and discharge-planning service [21,22].

### The geriatric consultation

**Figure 2:**
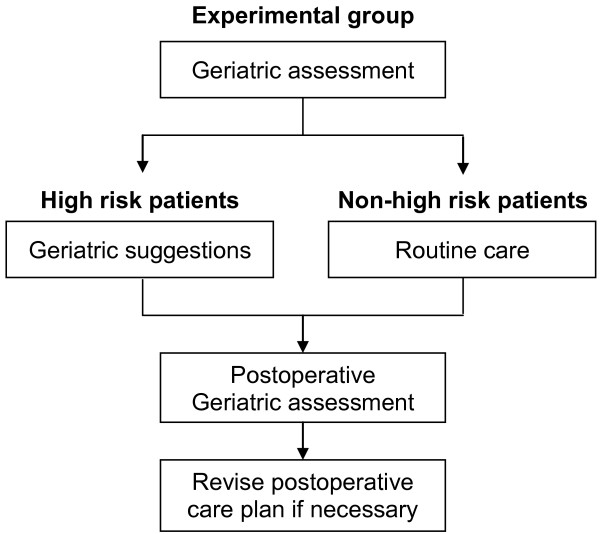
**Geriatric assessment**. Geriatric assessment flow chart.

Unlike routine care that provides no geriatric assessment, the intervention program offered geriatric consultation that was delivered by a geriatric nurse and a geriatrician. Before surgery, the geriatric nurse contacted the patient and completed the initial assessment to obtain medical and fall history, vital signs, physical examination, physical and cognitive functional assessment, nutritional status, preoperative risk assessment, current medications, and comorbidities. This information was reviewed by the geriatrician, who conducted geriatric assessments. The geriatric assessment was conducted for all subjects in the experimental group, and clinical suggestions were made for patients ≥ 80 years old, with high operative risk, poor nutritional status, cognitive impairment or disorientation, or those with unstable comorbid conditions (Figure 2). Based on this assessment, the geriatrician made suggestions to the surgeon regarding time of surgery, antibiotics and thromboembolic prophylaxis, postoperative nutritional management, urinary tract management, and delirium management/prevention. The geriatrician's suggestions were generally followed except the use of anticoagulants for thromboembolic prophylaxis underutilization rates. On the first day after surgery, the geriatric nurse visited the patient to assess for signs of delirium, pain, and postoperative complications. Based on this assessment, the geriatric nurse revised suggestions for the postoperative care plan to the surgeon. Each pre-surgical nursing assessment and geriatrician visit lasted around 60 minutes and the post-surgical nursing visit lasted 30 minutes.

### The rehabilitation program

Unlike routine care in which postoperative rehabilitation did not start until 2-3 days following surgery and no in-home rehabilitation was provided, the rehabilitation program in this study emphasized providing early postoperative rehabilitation and in-home rehabilitation. This rehabilitation program was delivered by the geriatric nurse, physical therapist, and rehabilitation physician. Both the in-hospital and in-home components of the rehabilitation program contained a hip fracture-oriented intervention and a general intervention for deteriorated physical fitness. The hip fracture-oriented rehabilitation emphasized pain relief, range of motion, muscle strength and endurance, proprioceptive enhancement, and balance challenges. The general intervention for deteriorated physical fitness rehabilitation emphasized exercises to increase physical fitness, including aerobic capacity, anaerobic capacity, muscle strength and endurance, and flexibility.

During hospitalization, rehabilitation started on the first day post-surgery and was delivered once a day by the geriatric nurse. According to patient's condition, the exercise protocol progressed from ankle exercises in bed to knee and hip joint flexion and extension exercises, to walking, and then climbing up and down stairs using a walker. During this period, around 4 rehabilitation sessions were provided by the geriatric nurse. In addition, the physical therapist made 2 visits to assess the patient's condition and provide rehabilitation sessions. The rehabilitation physician also made one visit to provide consultation.

For in-home rehabilitation, according to the patient's condition, the exercise protocol emphasized ankle dorsiflexion with knee extension, isometric full knee extension, gently bouncing vertical jump with knee semiflexed and foot on the floor, and ball rolling activities to enhance proprioception. During the first month, in-home rehabilitation was delivered by nurses once per week. During the second and third months, in-home rehabilitation was delivered by nurses once every 2 weeks. In addition, the physical therapist conducted one assessment within the first week after discharge, and at the third week and third month after discharge.

### Discharge planning

In addition to the geriatric consultation and rehabilitation components, a discharge service component was delivered by geriatric nurses. Unlike routine care, in which discharge planning is not provided to all patients and does not include home assessment, the geriatric nurse in this study assessed patients at discharge for caregivers' competence, resources, family function, patient self-care ability, patient and family caregiver needs for continuing health and social services, and made necessary referrals during hospitalization. The geriatric nurse also made a home visit before discharge to assess the home environment and suggested environmental modifications. The nurse also made phone calls to remind patients about follow-up visits to clinics.

### Measurement

#### Health-related quality of life (HRQOL)

To understand the impact of hip fracture on general health status, including physical symptoms, function, and emotional dimensions of health, rather than a specific aspect of health, a generic HRQOL measure was selected [16]. Generic HRQOL was measured by the Taiwan version [30] of the widely used SF 36 [31,32], which would allow further comparison among patients across different countries and/or with different diseases [33,34]. The SF-36 consists of 36 items representing eight generic health concepts: physical functioning (PF), role disability due to physical health problems (RP); bodily pain (BP); vitality (energy/fatigue) (VT); general health perceptions (GH); social functioning (SF); role disability due to emotional problems (RE); and general mental health (MH). For each scale, reverse items are recoded, the simple algebraic sums are computed, and the raw scale scores are transformed to a 0 to 100 scale. The higher the final score, the better the implied HRQOL. In addition, the SF-36 included one item on self-reported health transition (HT), which asked respondents to rate on a 5-point scale the amount of change in their general health compared to 1 year earlier. The higher the score, the more respondents believe that their general health is worse now than 1 year ago. Scores of this item were not transformed. Good validity and reliability of the SF-36 have been reported for the US elderly population [33-35]. The responsiveness of the SF-36 to assess hip-fracture outcomes has been established [4,36].

The SF-36 Taiwan version was translated and demonstrated good reliability and validity in a healthy adult sample [37,38]. The SF-36 Taiwan version is identical to the original SF-36. The reliability and validity of the SF-36 have been examined and established in elderly persons with hip fracture in Taiwan [30].

#### Pre-fracture self-care ability

The pre-fracture self-care ability of hip-fractured elders was retrospectively assessed using the Chinese Barthel Index (CBI), which measures dependencies in eating, transferring, grooming, toileting, bathing, walking, climbing stairs, dressing, and bowel and bladder control [39]. Scores range from 0 (total dependence) to 100 (total independence). In this study, Cronbach's alpha of the CBI was 0.87, suggesting high consistency.

#### Ethical considerations

This study was in compliance with the Helsinki Declaration and local legislation. Before data were collected, the study was approved for human subject research by the study hospital (Institutional Review Board, Chang Gung Memorial Hospital; approval number 89-25). Participants gave informed consent to participate in the study.

#### Procedure

Subjects were recruited from the ER by research assistants who screened the list of ER admissions twice a day for patients who met the inclusion criteria. Those who agreed to participate were randomly assigned right away (before surgery) to either an experimental or control group by the flip of a coin. Coin flipping was done by a neutral third party not involved in delivering the intervention or assessing outcomes. Subjects in the experimental group then received routine hospital care plus the intervention program, while subjects in the control group received only routine hospital care. All subjects were then assessed for HRQOL at 1, 3, 6, and 12 months after discharge. Due to the large proportion of illiterate participants (48%), data were collected during face-to-face interviews by research assistants reading the questionnaire aloud and recording participants' responses.

### Statistical analysis

Differences in baseline characteristics between the experimental and control groups were assessed by two-sample t-tests or chi-square tests. Effects of the interdisciplinary intervention were evaluated using a generalized estimating equation (GEE) approach to account for correlations in repeated observations over time. For a given outcome variable, the GEE model includes the following predictors: treatment (1 = experimental group, 0 = control group), and three dummy variables representing measurements made at 3, 6, and 12 months after hospitalization (with 1 month after discharge as the reference). GEE analyses were carried out using SAS Win 8.0.

All analyses were undertaken according to the intention-to-treat approach [40]. Missing data due to attrition (i.e., mortality, loss to follow-up, and refusal to participate) after randomization were imputed using multiple imputation [41,42]. For instance, we imputed missing data on performance of ADLs for subjects who dropped out or refused to participate after randomization by using baseline data (e.g., age, gender, education, functional status before fracture, functional status at discharge, and repeated observations of HRQOL if available).

For those who died during the trial, no imputation was made after death except for one subject in the experimental group who died before assessment at the first month after discharge. This decision was based on the principle of intent to treat, i.e., all randomized subjects should be included in the analysis [40]. In addition, the imputed value can be regarded as the outcome shortly before death. Furthermore, it should be noted that GEE allows partial information to be used in the analysis, i.e., data obtained before subjects' death can still contribute to estimating parameters.

Three complete data sets were imputed using NORM software developed by Schafer [43], and each set was analyzed. Estimates were then averaged across the three imputations to derive a single point estimate.

## Results

### Subjects' baseline characteristics

Of the 162 participants in the final sample, the majority (68.5%) were female, with an average age of 78.16 years (SD = 7.76). Around half the participants were married (51.9%) and illiterate (48.8%), 63% received internal fixation, and 37% received arthroplasty. The mean pre-fracture CBI was 96.08 (SD = 6.47), representing good independence in performing ADLs, and 84.6% could walk independently before the fracture. The experimental and control groups did not differ significantly in baseline characteristics (i.e., gender, age, marital status, education, type of surgery, pre-fracture performance of ADLs, body mass index [BMI], and walking ability) or timing of surgery and 1-year mortality (Table 1). Eighty-seven hip fractures were treated with internal fixation and followed-up for 1 year. Seven fractures failed to heal, and the union rate was 91%. However, only 80% of patients regained ambulatory ability. Fifty-one hip fractures were treated with arthroplasty and followed-up for 1 year. However, 90% of patients regained ambulatory ability. Two patients' hip prostheses were dislocated (2/51) and reduced with a closed technique [44]. The two groups were similar in weight bearing status, and were encouraged to ambulate with protected weight bearing for 3 months.

**Table 1:** Demographic characteristics of hip-fractured elders in the experimental and control groups

Characteristic	Experimental group (*n *= 80)	Control group (*n *= 82)	***P***^**a**^
Age (years), mean ± *SD*	77.36 ± 8.19	78.94 ± 7.28	0.20
Gender, *n *(%)			1.00
Female	55 (68.8)	56 (68.3)	
Male	25 (31.3)	26 (31.7)	
Marital status, *n *(%)			0.40
Single	1 (1.3)	0 (0)	
Married	38 (47.5)	46 (56.1)	
Widowed	40 (50)	36 (43.9)	
Divorced	1 (1.30)	0 (0)	
Educational background, *n *(%)			0.66
Illiterate	41 (51.3)	38 (46.3)	
Primary school	22 (27.5)	30 (36.6)	
High school	10 (12.5)	8 (9.8)	
College or above	7 (8.8)	6 (7.3)	
Time between fracture and surgery, *n *(%)			0.11
< 24 hours	28 (35.0)	35 (42.7)	
≥ 24 to ≤ 48 hours	15 (18.8)	22 (26.8)	
> 48 hours	37 (46.3)	25 (30.5)	
Type of surgery, *n *(%)			0.15
Internal fixation	55 (68.8)	47 (57.3)	
Arthroplasty	25 (31.3)	35 (42.7)	
Length of hospital stay (days), mean ± *SD*	10.12 ± 3.53	9.63 ± 4.83	0.14
Patients independent in walking ability,^b ^*n *(%)	68 (85)	69 (84.1)	1.00
Pre-fracture performance of ADLs,^c ^mean ± *SD*	95.94 ± 6.56	96.22 ± 6.41	-0.78
1-year mortality, *n *(%)	4 (5)	6 (7.2)	0.54
Body mass index (BMI), mean ± *SD*	22.05 ± 3.80	22.94 ± 3.80	0.22

^a^Determined by chi-square test

^b^Scores determined by Chinese Barthel Index, ranging from 0 (total dependence) to 100 (total independence).

^c^ADLs = activities of daily living; scores determined by CBI.

### Outcome comparison

**Figure 3:**
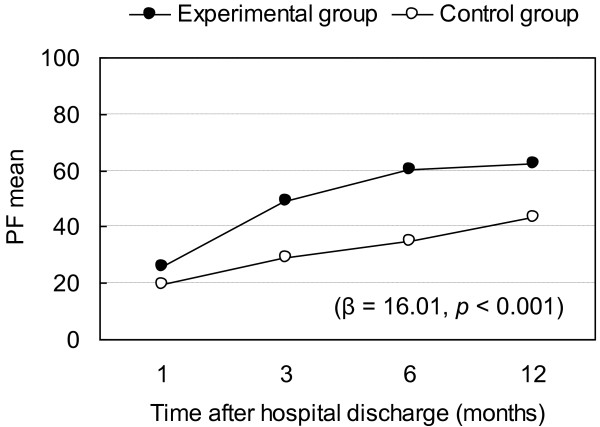
**Changes in Physical function (PF) and regression coefficient (p-value) of intervention effect**. Physical function (PF) at different time points and regression coefficients (p-value) for intervention effect on physical function.

**Figure 4:**
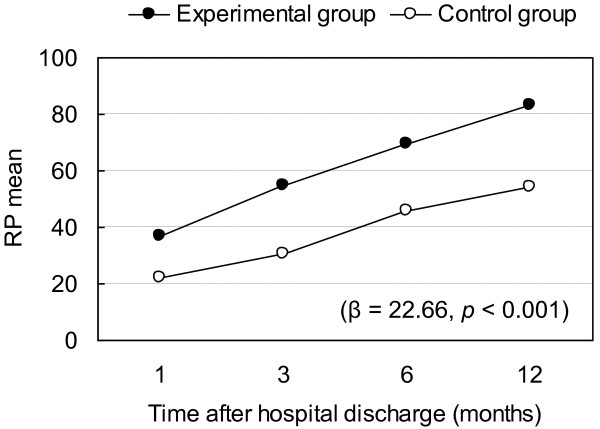
**Changes in Role physical (RP) and regression coefficient (p-value) of intervention effect**. Role physical limitations due to physical health problems (RP) at different time points and regression coefficients (p-value) for intervention effect on role physical.

Regression coefficients for overall effects by time and intervention are presented in Table 2. Outcomes for the experimental and control groups are compared according to the GEE approach using the first month and control group as baseline (Table 3). The intervention had a significant effect on subjects' bodily pain, vitality, mental health, physical function, and role physical (Table 2). After controlling for time, the experimental group had significantly better overall outcome in bodily pain (β = 9.38, p = 0.002), vitality (β = 9.40, p < 0.001), mental health (β = 8.16, p = 0.004), physical function (β = 16.01, p < 0.001), and role physical (β = 22.66, p < 0.001) than the control group at any time point during first year after discharge (Tables 2 and 3). β can be interpreted as representing the intervention effect on variables of HRQOL over the 12-month period after hospital discharge. In other words, the benefits of the interdisciplinary intervention program on bodily pain, vitality, mental health, physical function, and role physical lasted beyond 3 months after discharge. Furthermore, the benefits of the interdisciplinary intervention appeared to be greater for physical-related health outcomes such as physical function (Figure 3) and role physical (Figure 4).

**Table 2:** Regression coefficients of overall effects: time and intervention

	Time after discharge (months)			
		
Outcome variables	3	6	12	Group
Bodily pain (BP)	7.49^†^	8.65^‡^	12.65^‡^	9.38^†^
General health perceptions (GH)	-1.11	-3.30	-4.73	3.58
Vitality (energy/fatigue) (VT)	2.28	3.92	0.65	9.40^‡^
Social functioning (SF)	9.87^†^	15.71^‡^	17.47^‡^	5.62
Role limitations due to emotional problems (RE)	12.25^†^	11.36^‡^	17.42^‡^	3.08
General mental health (MH)	1.62	4.24*	1.80	8.16^†^
Physical functioning (PF)	14.55^‡^	21.37^‡^	25.37^‡^	16.01^‡^
Role limitations due to physical health problems (RP)	12.14*	26.25^‡^	37.18^‡^	22.66^‡^

Using 1-month data (after discharge). Group: using control group as baseline.

*p < 0.05; ^†^*p *< 0.01; ^‡^*p *< 0.001

**Table 3:** The outcome comparisons at different time point

	Mean (*SD*)		
		
Quality of life subscales	Experimental group	Control group	*P *value
Bodily pain (BP)			< 0.01
At 1^st ^month	67.70 (26.66)	59.32 (25.86)	
At 3^rd ^month	76.39 (22.91)	65.43 (25.88)	
At 6^th ^month	76.99 (23.44)	68.73 (27.78)	
At 1^st ^year	81.20 (22.73)	70.93 (26.94)	
General health perceptions (GH)			0.25
At 1^st ^month	48.21 (24.38)	50.22 (25.33)	
At 3^rd ^month	52.74 (24.29)	46.01 (24.53)	
At 6^th ^month	50.91 (25.05)	44.36 (24.06)	
At 1^st ^year	48.03 (26.81)	44.15 (22.82)	
Vitality (energy/fatigue) (VT)			< 0.001
At 1^st ^month	57.91 (24.50)	50.89 (23.45)	
At 3^rd ^month	63.87 (19.91)	51.93 (18.54)	
At 6^th ^month	64.37 (20.18)	54.71 (17.57)	
At 1^st ^year	60.86 (19.18)	51.32 (17.53)	
Social functioning (SF)			0.09
At 1^st ^month	51.30 (28.74)	48.87 (30.34)	
At 3^rd ^month	66.36 (27.62)	57.01 (26.93)	
At 6^th ^month	72.41 (28.42)	65.70 (28.56)	
At 1^st ^year	72.57 (28.17)	67.44 (27.87)	
Role limitations due to emotional problems (RE)			0.39
At 1^st ^month	72.34 (39.89)	71.07 (40.76)	
At 3^rd ^month	84.31 (33.36)	84.76 (29.40)	
At 6^th ^month	86.81 (28.83)	82.05 (33.76)	
At 1^st ^year	92.89 (19.98)	87.36 (28.35)	
General mental health (MH)			< 0.01
At 1^st ^month	61.44 (24.85)	54.06 (21.39)	
At 3^rd ^month	64.05 (21.25)	56.75 (20.55)	
At 6^th ^month	67.86 (20.28)	58.32 (20.09)	
At 1^st ^year	64.52 (19.03)	55.81 (18.70)	
Physical functioning (PF)			< 0.001
At 1^st ^month	26.13 (22.42)	19.80 (21.32)	
At 3^rd ^month	49.12 (29.57)	29.12 (24.56)	
At 6^th ^month	60.30 (28.02)	35.00 (24.58)	
At 1^st ^year	62.19 (28.08)	43.50 (28.47)	
Role limitations due to physical health problems (RP)			< 0.001
At 1^st ^month	36.76 (38.68)	22.13 (38.12)	
At 3^rd ^month	54.62 (40.58)	30.38 (36.28)	
At 6^th ^month	69.59 (37.33)	45.76 (40.78)	
At 1^st ^year	82.96 (28.96)	54.23 (40.04)	

*P *values were obtained from GEE models, after controlling for time.

For the time effect, bodily pain, social function, role emotional, physical function, and role physical were significantly better at the third, sixth, and twelfth months after discharge than at the first month after discharge. Mental health at the sixth month after discharge was significantly better than at the first month after discharge (Table 2).

## Discussion

As we hypothesized, the benefits of the interdisciplinary intervention program on HRQOL lasted throughout the first year after discharge. The results of this study expand those of previous studies showing that the treatment effects of more intensive, home-based rehabilitation programs or multidisciplinary programs for hip-fractured elders can be maintained up to 1 year after discharge, especially for physical-related functioning [9-12,14]. Similar to our findings, quality of life has previously been found to improve as a result of intervention programs for hip-fractured elders [14,15]. Our study further describes the trends in treatment effects on different dimension of HRQOL. Our multidisciplinary intervention program for elders in Taiwan with hip fracture significantly improved their bodily pain, vitality, social function, mental health, physical function, and role physical more than those of the control group at any time point during first year after discharge. It is difficult to separate the treatment effects for different components of this intervention program. Nonetheless, the rehabilitation combined with the geriatric consultation's clinical suggestions for high-risk patients might have improved their mental and physical function [45]. This possibility is supported by improved outcomes after hip fracture in a systematic review of multidisciplinary interventions including geriatric evaluation and management [46].

We also noticed that the trends in treatment benefits appeared to be larger for physical health-related outcomes such as physical function and role physical than other dimensions of HRQOL. The clinical significance of these differences in HRQOL outcomes can be assessed by the minimally important difference (MID), which indicates the smallest difference in score for the domain of interest that patients perceive as beneficial and mandating a change in the patient's management. A MID of 5 is suggested for the SF-36 [47,48]. The differences in BP, VT, and GH scores between the experimental and control groups at different time points were all greater than 7, indicating clinical significance. In particular, the differences in PH and RP scores at many time points were close to or greater than 20, indicating the magnitude of the intervention's positive effect.

To further assess the proportion of patients whose SF-36 summary score improved by 5 units from baseline to any time point during the 12-month period, we calculated the number needed to treat (NTT) [49] to achieve, on average, 1 patient with improved HRQOL for each scale. We found that the NTT for PH = 7.0, RE = 4.1, BP = 13.0, VT = 11.8, and GH = 6.4, SF = 7.0, RP = 13.2, and MH = 58.8. These numbers are consistent with a prior report that the physical functioning dimension of hip-fractured elders' HRQOL was poorer after discharge than mental/social dimensions [4]. Therefore, physical functioning might have a greater potential to be improved by treatment. Another possible reason for the apparent improvement in performance of physical function is that the physical function subscale of the SF-36 Taiwan version shows the best responsiveness to clinical changes [30].

Early surgery (within 48 hours of admission) after hip fracture was found in a systematic review of 52 studies to reduce hospital stay and possibly complications and mortality [50]. In our study 35% to 42.7% of subjects received surgery within 24 hours after fracture, close to a prior study [51]. Time between fracture and surgery did not differ significantly between the experimental and control groups in our study. This might due to timing of surgery depending largely on the time between hip fracture and patient admission, leaving little room for our intervention to intervene. Therefore, timing of surgery was treated in our study as a baseline characteristic, rather than an outcome variable.

Our study supports the long-term effects of an interdisciplinary intervention program for elders with hip fracture, but it had several limitations. First, the study design was single blinded only to subjects. This design was due to the technical difficulty of blinding assessors who would know right away from conversations with subjects which group they belonged to. To minimize the potential influence of bias, the personnel delivering the intervention and assessing outcomes were intentionally assigned different research duties. Second, this study lacked baseline measures for HRQOL before implementing the intervention program. However, the lack of significant differences in demographic characteristics and pre-fracture self-care ability of the experimental and control groups supports our assumption that the two groups had equivalent pre-intervention qualities of life and qualities of care. At the same time, our study's longitudinal design was able to demonstrate trends in changes for outcome variables and differences between the experimental and control groups, thus establishing the long-term effects of the treatment. It is also worth noting that although subjects were lost in the study period, the results obtained by intention-to-treat and on-protocol analyses were similar. Third, despite the similar size and demographics of the experimental and control groups, our method of randomization (coin flip) might have resulted in a dynamic bias [52] and can be considered a weakness of this study.

Finally, the generalizability of the study results might be limited by a sample selection bias in that our study criteria excluded elders with severe cognitive impairment and weak muscle power. Therefore, the study sample represents only 20% of the hip fracture population in the region. Thus, our sample may have had better function than the general population of elders with hip fracture in Taiwan. The effect of this intervention program can therefore only be generalized for hip-fractured elders without severe cognitive impairment and with adequate muscle power in their extremities. The estimated cost added by the intervention program to current routine care was $ 438 USD. The cost-effectiveness of this interdisciplinary program will be reported in detail in a separate paper.

## Conclusions

An interdisciplinary intervention with a geriatric hip-fracture program and discharge support component appeared to benefit elderly persons without severe cognitive impairment and with hip fracture in Taiwan by improving their HRQOL throughout the first year after discharge. The results of this study may provide a reference for health care providers in countries using similar programs with Chinese/Taiwanese immigrant populations.

## Competing interests

The authors declare that they have no competing interests.

## Authors' contributions

YS carried out the study and drafted the manuscript. JL conceived of the study design, participated in data analysis and helped to draft the manuscript. CW participated in designing and coordinating the study and helped to draft the manuscript. HC participated in designing and coordinating the study and helped to draft the manuscript. MC performed the statistical analysis and helped to draft the manuscript. All authors read and approved the final manuscript.

## Pre-publication history

The pre-publication history for this paper can be accessed here:

http://www.biomedcentral.com/1471-2474/11/225/prepub
